# Skeletal Muscle and Lymphocyte Mitochondrial Dysfunctions in Septic Shock Trigger ICU-Acquired Weakness and Sepsis-Induced Immunoparalysis

**DOI:** 10.1155/2017/7897325

**Published:** 2017-05-15

**Authors:** Quentin Maestraggi, Benjamin Lebas, Raphaël Clere-Jehl, Pierre-Olivier Ludes, Thiên-Nga Chamaraux-Tran, Francis Schneider, Pierre Diemunsch, Bernard Geny, Julien Pottecher

**Affiliations:** ^1^Hôpitaux Universitaires de Strasbourg, Hôpital de Hautepierre, Service de Réanimation Médicale, avenue Molière, 67098 Strasbourg Cedex, France; ^2^Université de Strasbourg, Fédération de Médecine Translationnelle de Strasbourg (FMTS), Faculté de Médecine, Institut de Physiologie, Equipe d'Accueil 3072 “Mitochondrie, Stress Oxydant et Protection Musculaire”, 11 rue Human, 67000 Strasbourg, France; ^3^Hôpitaux Universitaires de Strasbourg, Hôpital de Hautepierre, Service d'Anesthésie-Réanimation Chirurgicale, avenue Molière, 67098 Strasbourg Cedex, France; ^4^Institut de Génétique et de Biologie Moléculaire et Cellulaire (IGBMC), CNRS UMR7104, INSERM U964, Université de Strasbourg, Illkirch, France; ^5^Hôpitaux Universitaires de Strasbourg, Nouvel Hôpital Civil, Service de Physiologie et d'Explorations Fonctionnelles, 1 Place de l'Hôpital, 67091 Strasbourg Cedex, France

## Abstract

Fundamental events driving the pathological processes of septic shock-induced multiorgan failure (MOF) at the cellular and subcellular levels remain debated. Emerging data implicate mitochondrial dysfunction as a critical factor in the pathogenesis of sepsis-associated MOF. If macrocirculatory and microcirculatory dysfunctions undoubtedly participate in organ dysfunction at the early stage of septic shock, an intrinsic bioenergetic failure, sometimes called “cytopathic hypoxia,” perpetuates cellular dysfunction. Short-term failure of vital organs immediately threatens patient survival but long-term recovery is also severely hindered by persistent dysfunction of organs traditionally described as nonvital, such as skeletal muscle and peripheral blood mononuclear cells (PBMCs). In this review, we will stress how and why a persistent mitochondrial dysfunction in skeletal muscles and PBMC could impair survival in patients who overcome the first acute phase of their septic episode. First, muscle wasting protracts weaning from mechanical ventilation, increases the risk of mechanical ventilator-associated pneumonia, and creates a state of ICU-acquired muscle weakness, compelling the patient to bed. Second, failure of the immune system (“immunoparalysis”) translates into its inability to clear infectious foci and predisposes the patient to recurrent nosocomial infections. We will finally emphasize how mitochondrial-targeted therapies could represent a realistic strategy to promote long-term recovery after sepsis.

## 1. Introduction

Sepsis is a potentially lethal condition defined by life-threatening organ dysfunction caused by a dysregulated host response to infection [1]. The causal microorganism can either be bacterial, fungal, viral, or parasitical. Septic shock is a particularly severe form of sepsis associated with higher mortality rates. The classical definition described septic shock as the cardiovascular dysfunction associated with infection, if another cause of shock was ruled out [2, 3]. Severe sepsis referred to sepsis complicated by organ dysfunction and was an intermediary form of the disease whose severity lied between sepsis and septic shock. According to the recent Third International Consensus Definitions for Sepsis and Septic Shock (Sepsis-3), septic shock can be defined using the clinical criteria of arterial hypotension (requiring vasopressor to maintain mean arterial blood pressure over 65 mmHg) associated with a serum lactate level greater than 2 mmol/L despite adequate fluid resuscitation [1, 4]. Under Sepsis-3 terminology, sepsis is clinically characterized by a suspected or documented infection and an acute increase of ≥2 Sepsis-Related Organ Failure Assessment [5] (SOFA) points (a proxy for organ dysfunction). The term “severe sepsis” now becomes superfluous and its use is no longer recommended. For the sake of consistency, sepsis, severe sepsis, and septic shock terminologies are used in the following review according to the publication date of the reference cited (either before or after 2016).

The combination of hypotension, vasopressor use, and lactate level greater than 2 mmol/L, defining septic shock under Sepsis-3 terminology, is associated with hospital mortality rates consistently higher than 40%. These prohibitive mortality rates remain roughly unchanged despite extensive fundamental research and on-going medical progress. Understanding the pathophysiology of septic shock-induced multiorgan failure (MOF) thus remains a prerequisite to improve its outcome.

However, fundamental events driving the pathological processes of septic shock-induced MOF at the cellular and subcellular levels remain controversial [6, 7]. Indeed, autopsy studies of patients dying in the intensive care unit (ICU) from septic shock-induced MOF reveal only marginal histological injury [8]. If macrocirculatory [9] and microcirculatory [10] dysfunctions undoubtedly participate in organ dysfunction at the early stage of septic shock, an intrinsic bioenergetic failure (termed* cytopathic hypoxia* [11]) perpetuates cellular dysfunctions [12]. Thereby, cells become unable to consume available oxygen due to mitochondrial shutdown [13], consisting of impaired electron transport chain [14] (ETC), sustained reactive oxygen species (ROS) generation [15], and increased apoptosis [16].

Sepsis-induced failure of* vital* organs (heart, kidney, and liver) threatens immediately patient's survival. However mid- and long-term recovery is also hindered by persistent dysfunction of* crucial* organs, traditionally considered as nonvital, such as skeletal muscle or peripheral blood mononuclear cells (PBMCs). Concomitant dysfunctions in skeletal muscles (either locomotive or respiratory) and PBMCs fuel a feed-forward paresis-infection cycle, which ultimately leads to death.

However, comprehensively addressing septic shock-induced skeletal muscle and PBMC dysfunctions remains an unmet need. Therefore, our goals are to focus on these specific points and to stress how and why cellular and subcellular failure impair ICU outcome in patients who overcome the acute phase of septic shock. The role of persistent mitochondrial dysfunction in skeletal muscles and PBMC is specifically scrutinized. To conclude, we emphasize how mitochondrial-targeted therapies could improve long-term recovery after septic shock.

## 2. Mitochondrial Homeostasis in Health

Mitochondria are ubiquitous powerhouses of eukaryotic cells and they provide organisms with adenosine triphosphate (ATP) by using a group of enzymes, gathered in the mitochondrial electron transport chain (ETC), which transform transmembrane electron potential (ΔΨ_m_) in biochemical energy. Besides baseline oxidative phosphorylation, spare respiratory capacity (SRC) represents the mitochondrial phosphorylation reserve, available to cope with an extra metabolic demand.

A small part of oxygen, not used for oxidative phosphorylation, leads to the mitochondrial production of ROS at complexes I and III through uncoupling [17]. Depending on the amount and duration of ROS production, oxidative stress may either play a protective or deleterious role on mitochondrial signalling [18–20]. Intramitochondrial level of ROS is tightly regulated by several antioxidant enzymes like manganese superoxide dismutase (SOD_2_) or glutathione oxidase [21].

Mitochondrial hormesis (otherwise named mitochondrial biogenesis) is a cellular programed process that adjusts energy production by synthesis of new organelles and regulation of interorganelle interactions. The mitochondrial response to stressors (including sepsis) is sharply orchestrated by peroxisome proliferator-activated receptor-*γ* coactivator 1*α* (PGC-1*α*). PGC-1*α* is a transcriptional coregulator, which both modulates nuclear-encoded mitochondrial enzymes and controls mitochondrial desoxyribonucleic acid (mtDNA) transcription after interacting with mitochondrial transcription factor A (Tfam) [22]. PGC-1*α* expression is finely tuned by the energetic balance of the cell [23]. Furthermore mitochondrial biogenesis depends on intact immune system such as functionally active Toll-like receptors (TLRs) since genetically engineered TLR-4 deficient mice cannot activate mitochondrial recovery [24].

Other functions of mitochondria include synthesis of steroid hormones [25], intracellular calcium homeostasis, and induction of cell death through both the apoptotic (release of cytochrome c [12]) and the necrotic (massive mPTP opening [26]) pathways.

## 3. Sepsis-Induced Mitochondrial Dysfunction and Organ Failure

Mitochondrial dysfunction encompasses multiple aspects of disturbed cellular homeostasis, including decreased ATP production, increased generation of ROS, calcium dysregulation, and mtDNA damage [28]. Considering that mitochondria are the main site of whole body oxygen consumption [22], their role has been evoked to explain oxygen misuse and “cytopathic hypoxia” during sepsis [12].

### 3.1. A Coordinated Succession of Events

#### 3.1.1. Initial Multiorgan Failure

In vitro, sepsis-associated systemic inflammation produces ROS and reactive nitrogen species (RNS) primarily within the cytosolic NADPH oxidase [29] but also in mitochondria through disruption of the ETC [30]. Both ROS and RNS trigger sepsis-induced strand damage of mitochondrial DNA and induce nitrosylation [31] of mitochondrial ETC proteins.

Due to its vicinity of the ETC and its lack of protection by histones, mitochondrial DNA is very susceptible to oxidative and nitrosative stress [32, 33]. Damaged mtDNA translates into defective mitochondrial respiratory complexes, perpetuating cellular bioenergetic crisis and increased ROS production. Moreover, fragmented mtDNA can escape the mitochondrial matrix through intermittent opening of the mPTP and induce both intracytosolic and systemic signalling [33]. Indeed, mtDNA acts as a Danger-Associated Molecular Pattern (DAMP) in animal models of sepsis [34] and triggers both intracellular inflammasome activation (through NOD-like receptor family, pyrin domain containing 3, NLRP3 [35]) and remote organ injury (through Toll-like receptor 9 (TLR9) binding [36]).

In vitro [37], in animal models [38], prolonged inflammation induces decreased mitochondrial activity through increased nitric oxide (NO) levels and superoxide anion release, which combine to form the highly reactive peroxynitrite. Decreased mitochondrial activity is also worsened by inflammation-triggered ROS-mediated reduced gene transcription of mitochondrial-targeted genes. Indeed, dysfunctional ETC, reduced ATP levels, and reduced number of mtDNA copies following LPS exposure in mice can be abrogated with either overexpression of mitochondrial superoxide dismutase, iNOS inhibition, or peroxynitrite scavenger [39].

**Figure 1:**
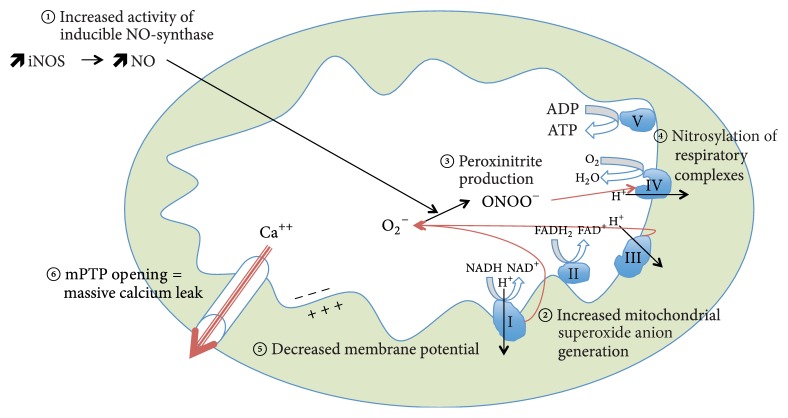
Succession of events leading to sepsis-induced mitochondrial dysfunction. (1) Increased activity of inducible NO-synthase. (2) Increased mitochondrial superoxide anion generation. (3) Production of peroxynitrite. (4) Nitrosylation of respiratory chain complexes. (5) Decreased membrane potential. (6) Opening of mitochondrial transition pore. Greek numbers refer to mitochondrial complexes. iNOS: inducible nitric oxide synthase; NO: nitric oxide; O_2_^−^: superoxide anion; ONOO^−^: peroxynitrite; O_2_: oxygen; ADP: adenosine diphosphate; ATP: adenosine triphosphate; H^+^: proton; FADH_2_: reduced flavin-adenine dinucleotide; FAD^+^: oxidized flavin-adenine dinucleotide; NADH: reduced nicotinamide adenine dinucleotide; NAD^+^: oxidized nicotinamide adenine dinucleotide; Ca^++^: ionized calcium; mPTP: mitochondrial permeability transition pore.

In humans, it is hypothesized that dysfunctional mitochondrial respiratory chain results in reduced ATP turnover and metabolic shutdown. When metabolic shutdown is sufficiently intense to prevent adequate production of ATP, cells can no longer maintain energy availability for essential cellular functions and go into either necrotic or apoptotic cell death pathways [40], triggering organ failure and death (Figure 1).

However, the initial multiorgan metabolic shutdown may be revocable, since most organs recover when the septic patient overcomes his infectious episode [41]. At the subcellular level, decreased activity of the mitochondrial ETC in human sepsis may be reversible as well. Indeed, endothelial cells perfused in vitro with serum from septic shock patients show a 40% decrease in mitochondrial respiration, which is restored with both a NOS inhibitor and a blocker of the poly(ADP-ribose) synthase pathway [42]. Conversely, the nitrosylation of mitochondrial ETC complexes might become persistent when the mitochondrial glutathione pool is oxidized [31, 43]. Even if human data are scarce [43] many experimental findings converge to persistent S-nitrosylation of complex I as a trigger for persistent ETC dysfunction, increased superoxide production [44], energy failure crisis, mPTP opening, and release of proapoptotic factors [43, 45].

#### 3.1.2. Subsequent Activation of Mitochondrial Hormesis

**Figure 2:**
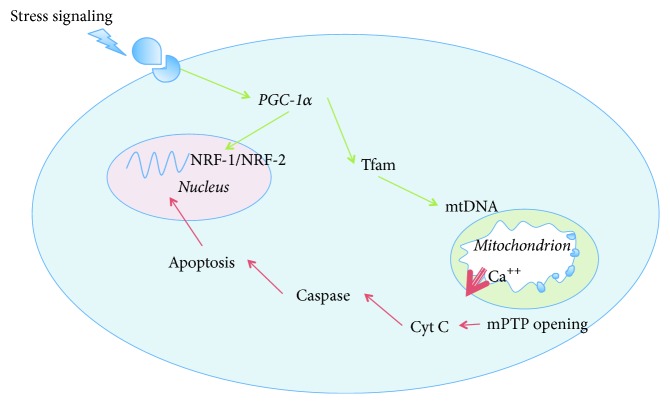
Nucleus-mitochondria crosstalk for apoptosis and mitochondrial hormesis induction: key role of PGC-1*α*, Tfam, and NRF-1/2. (i) Activation of caspase-3 and caspase-7 after mPTP opening leading to apoptosis. (ii) Activation of mitochondrial hormesis in response to stress stimulation (i.e., *β*-adrenergic). PGC-1*α* plays a pivotal role for transcription of both nuclear and mitochondrial genes leading to increased mitohormesis. PGC-1*α*: peroxisome proliferator-activated receptor-*γ* coactivator 1*α*; NRF-1/2: nuclear respiratory factor 1/2; Tfam: mitochondrial transcription factor A; mtDNA: mitochondrial desoxyribonucleic acid; Ca^++^: ionized calcium; mPTP: mitochondrial permeability transition pore; Cyt c: cytochrome c.

If the organism (either animal or human) survives, as septic shock recovers, prolonged exposure to lower levels of NO activates mitochondrial biogenesis that promotes mitochondrial proliferation and a progressive increase in cell metabolism [46] (Figure 2). Consecutive to the activation of the mitochondrial biogenesis program, expression of NRF-1, NRF- 2, PGC-1*α*, and Tfam accompanies restoration of mtDNA copy number [22]. This has elegantly been demonstrated by Bartz et al. in a murine model of* Staphylococcus aureus* sepsis [47].

The switch from mitochondrial metabolic shutdown to mitochondrial biogenesis is finely regulated and orchestrated by many factors that ultimately depend on sepsis severity and resilience of the organism [48] such as age, comorbidities, and genetic factors (e.g., mitochondrial haplotype in man [49]). The resulting signals will determine mitochondrial mass, number, size, distribution, and phenotype across cell types including skeletal muscle cells [50] and PBMCs [16] in ICU patients. Some mitochondrial haplotypes seem to convey an evolutionary benefit. For instance, in Newcastle upon Tyne (UK) haplogroup H was a strong independent predictor of better outcome during severe sepsis in 150 consecutive patients, conferring a 2.12-fold (95% CI 1.02–4.43) increased chance of survival [49].

### 3.2. Sepsis-Induced, Mitochondrial-Driven ICU-Acquired Muscle Weakness in Critically Ill Patients

ICU-acquired weakness is now a recognized clinical consequence of skeletal muscle mitochondrial dysfunction [51], which occurs simultaneously in respiratory (diaphragm, intercostal) and locomotive* (vastus lateralis)* muscles. ICU-acquired weakness undoubtedly contributes to worse mid- and long-term prognosis in patients with septic shock [52]. As recently underlined by Batt et al., ICU-acquired weakness has become a priority research focus in critical care [53]. For instance, ICU-acquired, sepsis-induced diaphragmatic dysfunction was strongly associated with increased ICU and hospital mortality [54]. Moreover, ICU-acquired limb weakness is an important patient-centered outcome with clear implications for quality of life [52]. In a prospective multicentre study, Ali et al. established that ICU-acquired weakness was independently associated with hospital mortality (odds ratio [OR], 7.8; 95% confidence interval [CI], 2.4–25.3; *P* < 0.001) even after adjustment for severity of illness [55]. Handgrip dynamometry providing a concise measure of global strength was independently associated with hospital mortality (OR, 4.5; 95% CI, 1.5–13.6; *P* < 0.007). Muscular weakness and functional disability can persist as long as five years after critical illness [56].

#### 3.2.1. Sepsis-Induced, Mitochondrial-Driven ICU-Acquired Diaphragm Weakness

As it extends ventilator-dependent duration, ICU-acquired diaphragm weakness may increase persistent functional limitation, the incidence of ventilator-associated pneumonia, and healthcare-associated morbidity.

In a murine model of septic shock, Zolfaghari et al. demonstrated that maximal force generation was reduced and fatigue accelerated in ex vivo diaphragm muscle strips from septic mice, together with lower mitochondrial Δ*ψ*m and phosphorylation capacity [57]. Sepsis-induced respiratory muscle proteolysis (mediated by calpain, caspase-3, and ubiquitin-proteasome system) may be exacerbated by mechanical ventilation as an additional mechanism for acquired diaphragm weakness [58] in both animal models [59] and clinical situations [60].

Even if clinical evidence of sepsis-induced direct diaphragm mitochondrial dysfunction is still lacking in man, mechanical ventilation was shown to induce profound defects in diaphragm mitochondrial biogenesis and cytochrome c oxidase content in ventilated brain-dead organ donors [60]. Thus, sepsis and mechanical ventilation may have synergistic deleterious effects on diaphragm mitochondrial function in ventilated septic shock patients.

#### 3.2.2. Sepsis-Induced, Mitochondrial-Driven ICU-Acquired Limb Weakness

Concerning limb muscles, deficits in both strength and size may exceed those seen in the diaphragm. For instance, Mofarrahi et al. reported reduced resistance to increased Ca^2+^ load and deeper biogenesis reduction in locomotor muscles (*soleus* and* tibialis anterior*) compared to diaphragm after a LPS challenge in mice [61]. Similar findings were reported among patients with sepsis [62].

Experimental findings suggest that prolonged and unopposed inflammatory processes during sepsis amplify TNF*α* production, whose catabolic effects on murine skeletal muscle trigger both decreased mitochondrial respiration and impaired biogenesis (through PGC-1*α* inactivation [61]). As such, sustained exposure of murine skeletal myotubes to TNF*α* induces reduced expression of oxidative phosphorylation subunits [63] in a NF*κ*-B-dependent manner. Some evidence also suggests that a circulating factor (either NO or/and IL_6_ and/or mtDNA) may be at work to explain widespread skeletal muscle protein loss (myosin breakdown, proteolysis) [64] and altered mitochondrial function [16, 43] in septic shock patients.

However, based on recent data from our team and others, implication of a circulating plasmatic factor remains unclear to account for muscular mitochondrial ETC decreasing activity in both experimental models and septic shock patients. For instance, in a short-term CLP-induced murine septic shock model, ex vivo mitochondrial assessments showed neither reduced enzymatic activities, altered mitochondrial respiration, nor reduced tolerance to calcium loading [65]. Moreover, exposure of rodents skeletal muscle mitochondria with plasma of septic shock patients had no significant effect on muscle mitochondrial respiration [66].

Pioneering studies from Mervyn Singer's laboratory established that mitochondrial ETC was severely impaired in skeletal muscle biopsies from septic shock patients [43], harvested within 24 h of ICU admission. Furthermore, mitochondrial complex I activity had a significant inverse correlation with both shock severity and NO production and had a significant positive correlation with concentrations of intracellular antioxidant concentration and ATP content. Similarly, in a recent human quadriceps comparative study, Jiroutková et al. confirmed the dysfunction in mitochondrial respiration, showing functional impairments and quantitative defects of respiratory complexes in muscle of septic patients compared to controls [67]. It must be stressed that in both Brealey et al.'s[43] and Jiroutková et al.'s [67] clinical studies, muscle samples were collected more than 24 h after sepsis onset, differing in this way from many experimental “acute” models (including ours [65]) and potentially explaining contrasting results [68].

Using microarray analysis the Singer's team consequently demonstrated that septic shock survivors were able to activate mitochondrial hormesis pathways (PGC-1*α* and SOD_2_) that counteract early decrease in mitochondrial ETC [50]. On the opposite, sepsis-survivors with protracted critical illness and ICU-acquired weakness still demonstrate a 50% reduction of oxidative phosphorylation in their* vastus lateralis* muscles [67]. Pharmacological induction of mitochondrial hormesis might therefore be an interesting way to overcome both the acute septic phase and the subsequent long-lasting ICU-acquired weakness and therefore stands for a promising research to promote muscle recovery in critical illness.

### 3.3. Sepsis-Induced, Mitochondrial-Driven Immunosuppression

As mitochondria used to be ancient endosymbiotes, defence mechanisms from the host immune response directed towards the non-self also recognize molecular patterns shared by mitochondria (containing many DAMPs) and pathogen agents (PAMPs) [69]. The immune system and its interactions with extracellular mitochondrial components (mtDNA [70], formyl peptide [36], and Tfam [71]) thus play a crucial role in inducing both initial widespread inflammatory response (which contributes to mitochondrial dysfunction through oxidative stress) and later mitochondrial recovery and hormesis.

Initially, exhaustion of the immune system leads to immunoparalysis. Potential mechanisms of immune suppression in patients with septic shock include shift from proinflammatory to anti-inflammatory phenotype, lymphocyte mitochondrial energy and apoptosis-induced depletion of CD4 positive cells and dendritic cells [8]. For instance, in spleens removed after the death of septic shock patients, Hotchkiss et al. observed that the duration of the septic episode was significantly associated with the depletion of B cells and CD4 T cells [72].

#### 3.3.1. Sepsis-Induced, Mitochondrial-Driven Lymphocyte Apoptosis

Septic shock is classically associated with lymphopenia [73], which appears at the early stage of the disease [74] and contributes to the Compensatory Anti-Inflammatory Response Syndrome (CARS). CARS is now considered to be developing in parallel with the proinflammatory response, as early as sepsis onset. This “compensatory” anti-inflammatory response leads to a deep, sepsis-induced immunosuppression, which may be responsible for late deaths [75]. Lymphopenia is part of CARS and results from apoptosis of roughly all classes of lymphocytes, especially B and CD4^+^ T cells [72]. The triggers of this apoptosis seem to be multiple, involving death receptor as well as mitochondrial-mediated apoptosis.

In a murine sepsis model of caecal ligation and puncture, Chang et al. have shown that downregulation of genes involved in mitochondrial-mediated apoptosis (*Bim*−/− mice) provided a decrease in lymphocytes apoptosis and a better mice survival [76]. Mitochondrial-mediated apoptosis has also been shown to be enhanced by the rate of circulating histones, which induce mitochondrial injury in vitro [77].

In humans, involvement of mitochondrial-mediated apoptosis in sepsis has been evidenced by a caspase-9-mediated profound progressive loss of B and CD4+ T cells [72]. In 16 severe sepsis patients (13 of whom matching the updated septic shock definition), lymphocyte apoptosis resulted from both a downregulation of genes inhibiting mitochondrial-mediated apoptosis (*Bcl*-2 and* Bcl*-xl) and an upregulation of proapoptotic genes (*Bim*,* Bid,* and* Bak*) [78]. Moreover, pharmaceutically decreasing* Bak*/*Bcl*-2 and* Bax*/*Bcl*-xl ratios could prevent apoptosis PBMC in septic shock patients [79].

#### 3.3.2. Sepsis-Induced, Mitochondrial-Driven Impaired Lymphocyte Function

Lymphocyte mitochondrial dysfunction has been shown in sepsis and was ascertained by an early decrease in mitochondrial respiratory capacity. Garrabou et al. observed a 16% decrease in spontaneous cell oxygen consumption in PBMCs from severe sepsis patients. Concurrent apoptosis process was shown in septic patients by an elevated caspase-3 activity and by a much higher percentage of cells with depolarized mitochondria. No change in cell mitochondrial content was observed at that stage [16]. Japiassú et al. pointed out a 43% decrease in cell respiration, specifically associated with reduced ATP synthesis in immune cells of septic shock patients compared with controls. This reduced energy synthesis came along with a 49% reduction in F_1_F_0_ ATP-synthase activity in septic patients [80]. The same study found that reduction in ATP synthesis was significantly associated with organ failure and death, so that the degree of mitochondrial impairment was correlated with the clinical outcome.

Mechanisms leading to mitochondrial dysfunction in circulating lymphocytes remain unclear. In the vein of muscular mitochondrial dysfunction, the influence of septic plasma has been suggested. Indeed, Belikova et al. observed a lower response to ADP stimulation and an increased fraction of decoupling oxygen consumption in septic PBMC and in healthy PBMC after incubation in septic plasma, whereas septic PBMC incubated in healthy plasma partially recovered normal respiratory capacities [81]. Plasma factors involved in mitochondrial dysfunction may include NO, ROS [82], and mitochondrial fractions themselves (mtDNA, formyl peptide, and Tfam). Indeed, as previously described, increased mitochondrial ROS production induces mtDNA fragmentation and the release of fragmented mtDNA in both the cytoplasm and the extracellular space. Acting as a DAMP, mtDNA then binds TLR9 on neutrophil plasma membrane and induces both remote organ injury [83] and immunosuppression [84].

Functional alterations in PBMCs may also account for septic shock-induced immunosuppression. For instance, Weiss et al. demonstrated that mitochondrial SRC was reduced and uncoupled respiration was increased in septic shock children PBMCs compared to controls, suggesting that mitochondria within immune cells could not keep pace with an increase in energy demand [85]. If SRC is not sufficient to meet the sepsis-induced increase in metabolic demand, PBMCs may experience an energetic exhaustion, leading to immunoparalysis. Thus, mitochondrial energy failure may be, at least in part, responsible for the inability of the septic PBMCs to respond adequately to pathogen invasion.

#### 3.3.3. Functional Aspects of Lymphocyte Mitochondrial Biogenesis after Sepsis

**Figure 3:**
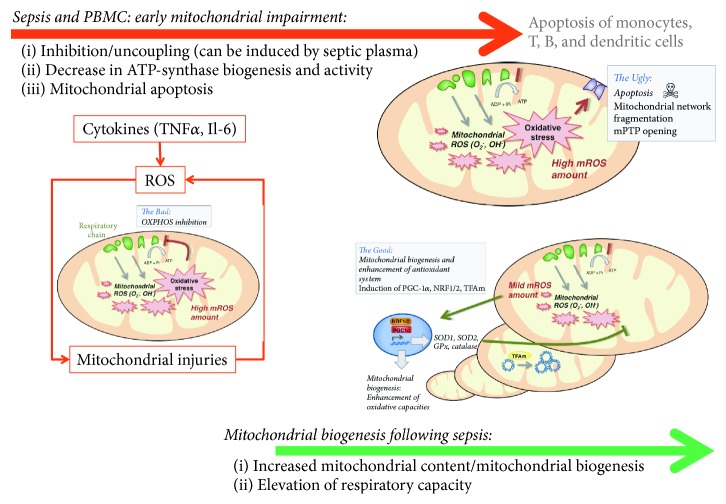
Time course of mitochondrial impairment and mitochondrial biogenesis following sepsis in peripheral blood mononuclear cells (PBMCs). TNF*α*: tumor necrosis factor *α*; Il-6: interleukin-6; ROS: reactive oxygen species; SOD: superoxide dismutase, GPx: glutathione peroxidase; NRF-1/2: nuclear respiratory factor 1/2; PGC-1*α*: peroxisome proliferator-activated receptor-*γ* coactivator 1*α*; Tfam: mitochondrial transcription factor A.* Adapted from Lejay et al. with permission* [27].

In septic shock survivors, mitochondrial recovery, and biogenesis have been suggested [24] (Figure 3). After an initial drop, Sjövall et al. observed subsequent increase in cell respiratory capacity in septic patients after admission in intensive care unit (ICU). As early as 48 h after ICU admission, baseline oxygen consumption had increased by 27% in septic compared to healthy PBMC [86]. At day 7, baseline oxygen consumption in septic cells had risen by 73% compared with control. These modifications were associated with enhanced mitochondrial biogenesis biomarkers: citrate synthase activity, mtDNA, and cytochrome c [86]. As a result, mitochondrial respiration, when expressed in relation to citrate synthase activity, displayed a 27% to 52% reduction. Increased oxygen consumption rather reflected an increased mitochondrial content than an empowered ETC.

Moreover, increase of mitochondrial biogenesis was associated with lymphocytes functional improvements, such as CD8+ T cell memory development in mice [87]. CD8+ T memory cells had a higher SRC (the extra capacity available in cells to produce energy in response to increased stress), correlated with mitochondrial biogenesis. Similar findings were found in paediatric septic shock patients by Weiss et al. [85].

These results suggest that initial mitochondrial dysfunction in sepsis would induce both quantitative and qualitative impairments of immune cells and that mitochondrial biogenesis may subsequently compensate for these deficiencies by an increased mitochondrial mass and a higher resilience to stressors.

### 3.4. Synergic Deleterious Effects of Sepsis-Induced, Mitochondrial-Driven Immunosuppression and Acquired Muscle Weakness

Even if a causative link still has to be demonstrated between sepsis-induced muscle weakness/immunoparalysis on the one hand and increased incidence of ventilator-acquired pneumonia on the other hand, many indices converge to a common pathophysiologic pathway. Indeed, sepsis-induced ICU-acquired muscle weakness triggers diaphragmatic dysfunction, weaning failure, and prolonged mechanical ventilation, which is associated with increased mortality when weaning is prolonged above 7 days [88]. In addition, patients suffering from ICU-acquired muscle paresis have a twice larger risk of recurrent respiratory failure [55]. ICU-acquired muscle weakness also results in significant atrophy of the pharyngeal, laryngeal, and accessory inspiratory muscles and triggers ICU-acquired swallowing disorders, reduced cough strength, and limited glottis clearance [89]. As dysphagia predisposes to microaspirations in extubated ICU patients, swallowing disorders are clearly associated with a composite outcome of pneumonia, reintubation, or death (adjusted odds ratio, 3.31 [1.78–4.56]; *P* < 0.01) [90].

Actually, swallowing disorders and reduced cough strength decrease airway clearance and increase the pathogen load (bacterial, viral, and fungal) to the lungs. If immune response effectors are also worn-out, pathogens can develop unrestrainedly. From the immunological point of view, severe persistent sepsis-induced lymphopenia was associated with increased development of secondary infections and death [91]. Sepsis-induced, mitochondrial-driven lymphocyte apoptosis triggers an increased uptake of apoptotic cells by surviving monocytes, macrophages, and dendritic cells, which cannot fight the remaining pathogenic organisms [92]. In this way, sepsis-induced mitochondrial-driven muscle dysfunction and sepsis-induced mitochondrial-driven immunosuppression fuel a feed-forward cycle, culminating in the development of ICU-acquired, healthcare-associated infections, particularly ventilator-associated pneumonias.

## 4. Mitochondrial-Targeted Therapies to Promote Mitohormesis in Skeletal Muscles and PBMC after Septic Shock

As mitochondrial dysfunction in skeletal muscle and PBMC potentially contributes to protracted weaning from mechanical ventilation and persistent infectious foci in patients recovering from septic shock, therapies promoting mitochondrial healing may favour recovery of both muscular functions and immunologic potency. Efforts should be made to focus on the good treatment in the right place and at the appropriate time.

### 4.1. Early Hemodynamic Resuscitation

Even if restricted oxygen uptake and global tissue hypoxia may trigger inflammatory cascade at the onset of sepsis [93] and if early goal-directed therapy (EGDT) improves biomarkers patterns [94] and patient outcome [9] in the early stages, the “window of opportunity” for EGDT to promote mitochondrial hormesis appears to be narrow and not reproducible in every setting [95]. Indeed, many studies report increased oxygen availability in resuscitated septic shock, in both experimental [96] and clinical [97] settings. Corrêa et al. observed that treatment delay beyond six hours after the onset of septic shock reduced skeletal muscle ATP content in swine [98]. Norepinephrine treatment in endotoxaemic pigs did not increase hepatosplanchnic flow, oxygen delivery, or consumption and did not improve the hepatic lactate extraction ratio. However, norepinephrine increased the liver mitochondria complex I-dependent and complex II-dependent respiratory control ratios [99]. The question as to whether EGDT could reduce mitochondrial dysfunction in skeletal muscles and PBMCs in septic shock patients still remains unresolved.

### 4.2. Substrate and Cofactor Provision

As complex I is predominantly damaged during sepsis [44], Protti et al. hypothesized that adding succinate, the specific complex II substrate would increase electron flow through the distal part of the ETC and improve mitochondrial oxygen consumption. In soleus muscles harvested from septic rats, the authors demonstrated that succinate provision bypassed the predominant sepsis-induced ETC inhibition (occurring at complex I) and restored oxygen consumption and ATP synthesis to nonseptic levels [100]. To our knowledge, this interesting pathway has not been yet translated to therapeutic strategies in septic shock patients.

The process of *β*-oxidation involves the breakdown of fatty acids to generate acetyl-CoA, NADH, and FADH_2_ and requires L-carnitine for the transport of long-chain cytosolic fatty acids into mitochondria for *β*-oxidation [101]. Van der Windt et al. suggest that drugs that target mitochondrial respiratory capacity in PBMCs (such as carnitine palmitoyl transferase) could hold promise as immunotherapeutics and might warrant further study for their ability to alter T cell responses.

### 4.3. Mitochondrial Antioxidants and ROS Scavengers

Resveratrol, a natural phenol within red grapes [102], exerts beneficial effects in experimental sepsis when administered either before or shortly after the septic insult [103]. Recent research has demonstrated that resveratrol activates silent mating type information regulator 2 homolog 1 (SIRT1) in mice, which is a key regulator of cellular defences and mitochondrial hormesis in response to metabolic stress [104]. Resveratrol has also been shown to downregulate the proinflammatory response and to have antioxidant properties in aging rats [103]. Mitochondrial biogenesis restores oxidative metabolism and thus enhances prosurvival during* Staphylococcus aureus* sepsis in mice [71].

However, some antioxidants may fail to induce mitohormesis, due to inadequate mitochondrial upload, and mitochondrial-targeted therapies hold promises. In mechanically ventilated mice, Picard et al. demonstrated that transgenic overexpression of a mitochondria-localized antioxidant (peroxiredoxin-3, downregulated in mechanically ventilated human diaphragms) was protective against ventilation-induced diaphragmatic dysfunction [60]. In skeletal muscle, melatonin (a powerful antioxidant) might counteract inducible mtNOS-triggered dysfunction of the ETC, as recently demonstrated in septic mice [105]. Other mitochondria-targeted lipophilic antioxidants like coenzyme Q_10_ improved mitochondrial biogenesis in LPS-treated mice by activating PGC-1*α* and Tfam [106]. One such approach covalently links biomolecules to lipophilic triphenylphosphonium cation (TPP+). Due to a positive charge, the molecules are driven by the mitochondrial membrane potential to accumulate solely in mitochondria [107]. In their murine septic shock experiment, Zang et al. tested a single, postinfection administration of mitochondria-targeted antioxidant (Mito-Vit-E). Interestingly, Mito-Vit-E provided mitochondria-specific antioxidant defense and improved mitochondrial respiratory function in the heart over time with sepsis, while cytosolic-targeted Vit-E had no such effect [107]. To date, no clinical study supports these encouraging preclinical findings.

Among research possibilities to confirm the key role of initial mitochondrial dysfunction in sepsis, antioxidants could therefore be used to improve mitochondrial work at an early stage of sepsis, in order to diminish mitochondrial-mediated ROS production and apoptosis. Besides, regulation of genes involved in lymphocytes mitochondrial-mediated apoptosis (e.g., Bcl-2) and TLR2-mitochondria axis deserve to be more studied [108].

## 5. Conclusion

The “tissue hypoxia” paradigm in sepsis has recently been challenged and should prompt research in the field of cytopathic hypoxia. In septic shock patients, a circulating factor (which could be nitric oxide, superoxide anion, peroxynitrite, or mtDNA) induces mitochondrial ETC dysfunction and increases apoptosis in both myocytes and PBMCs. These processes profoundly impair organ recovery and patient rehabilitation. Therapeutic induction of mitochondrial hormesis (increased mitochondrial mass and a higher resilience to stressors) may be a way to overcome the septic episode and might stand for a promising research to promote organ recovery in critical illness.

## List of Abbreviations

Akt: Protein kinase B
ATP: Adenosine triphosphate
CARS: Compensatory anti-inflammatory response syndrome
ΔΨ_m_: Mitochondrial transmembrane electron potential
ETC: Electron transport chain
EGDT: Early goal-directed therapy
FAD^+^: Oxidized flavin-adenine dinucleotide
FADH_2_: Reduced flavin-adenine dinucleotide
FCCP: Carbonyl cyanide-4-(trifluoromethoxy)phenylhydrazone
GSK-3*β*: Glycogen synthase kinase 3*β*
ICU: Intensive care unit
iNOS: Inducible nitric oxide synthase
MOF: Multiorgan failure
mPTP: Mitochondrial permeability transition pore
mtDNA: Mitochondrial desoxyribonucleic acid
NAD^+^: Oxidized nicotinamide adenine dinucleotide
NADH: Reduced nicotinamide adenine dinucleotide
NO: Nitric oxide
NLRP3: NOD-like receptor family, pyrin domain containing 3
NRF: Nuclear respiratory factors
PBMCs: Peripheral blood mononuclear cells
PGC-1*α*: Peroxisome proliferator-activated receptor-*γ* coactivator 1*α*
RNS: Reactive nitrogen species
ROS: Reactive oxygen species
SIRT: Sirtuin
SIRT1: Silent mating type information regulator 2 homolog 1
SOD_2_: Manganese superoxide dismutase
SOFA: Sepsis-Related Organ Failure Assessment
SRC: Spare respiratory capacity
Tfam: Mitochondrial transcription factor A
TLR: Toll-like receptor.
